# Microcalcification Discrimination in Mammography Using Deep Convolutional Neural Network: Towards Rapid and Early Breast Cancer Diagnosis

**DOI:** 10.3389/fpubh.2022.875305

**Published:** 2022-04-28

**Authors:** Yew Sum Leong, Khairunnisa Hasikin, Khin Wee Lai, Norita Mohd Zain, Muhammad Mokhzaini Azizan

**Affiliations:** ^1^Department of Biomedical Engineering, Faculty of Engineering, Universiti Malaya, Kuala Lumpur, Malaysia; ^2^Department of Biomedical Engineering, Center for Image and Signal Processing (CISIP), Faculty of Engineering, Universiti Malaya, Kuala Lumpur, Malaysia; ^3^Department of Electrical and Electronic Engineering, Faculty of Engineering and Built Environment, Universiti Sains Islam Malaysia, Nilai, Malaysia

**Keywords:** transfer learning, region of interest (ROI), intervention, machine learning, artificial intelligence

## Abstract

Breast cancer is among the most common types of cancer in women and under the cases of misdiagnosed, or delayed in treatment, the mortality risk is high. The existence of breast microcalcifications is common in breast cancer patients and they are an effective indicator for early sign of breast cancer. However, microcalcifications are often missed and wrongly classified during screening due to their small sizes and indirect scattering in mammogram images. Motivated by this issue, this project proposes an adaptive transfer learning deep convolutional neural network in segmenting breast mammogram images with calcifications cases for early breast cancer diagnosis and intervention. Mammogram images of breast microcalcifications are utilized to train several deep neural network models and their performance is compared. Image filtering of the region of interest images was conducted to remove possible artifacts and noises to enhance the quality of the images before the training. Different hyperparameters such as epoch, batch size, etc were tuned to obtain the best possible result. In addition, the performance of the proposed fine-tuned hyperparameter of ResNet50 is compared with another state-of-the-art machine learning network such as ResNet34, VGG16, and AlexNet. Confusion matrices were utilized for comparison. The result from this study shows that the proposed ResNet50 achieves the highest accuracy with a value of 97.58%, followed by ResNet34 of 97.35%, VGG16 96.97%, and finally AlexNet of 83.06%.

## Introduction

In 2020, World Health Organization (WHO) reported 2.3 million cases of breast cancer worldwide with over 685,000 fatalities, making it among the highest fatal diseases in the world. Although extensive efforts on breast cancer screening have shown promising results for early intervention, localizing breast lesions has remained a challenge. This is because detection of breast lesions on mammogram images heavily depended on the radiologist's skill (1), which proved to be time consuming, and at times lacked the accuracy and precision Thus, this factor poses a serious challenge onto rapid diagnosis process which in the case of breast cancer, late detection may prove terminal. Advancements and involvement of artificial intelligence (AI) in the healthcare sector have improved accuracy and assisted radiologists by minimizing the rates of false positives and false negatives during clinical diagnosis. Deep Convolutional Neural Networks (D-CNN), a subsidiary of AI, have advanced to the point where they can automatically learn from enormous picture data sets and detect abnormalities in mammograms such as mass lesions (2). D-CNN has quickly become the preferred approach for evaluating medical images to aid the early detection of breast cancer diseases, which resulted in a favourable prognosis and a higher percentage of survival (3, 4).

The presence of microcalcification during breast cancer screening is often missed due to its small size which is approximately 0.1–1.0 mm. In addition, it may be scattered and less visible to naked eyes due to the surrounding dense breast tissues. Different from microcalcification, breast lump has a relatively high predictive value for malignancy (5, 6). Calcifications may appear as white dots with specific patterns, size, density, and location on mammogram images, which might signify breast cancer or precancerous alterations in breast tissue (7). Even with visible calcifications, most lesions are not recalled immediately but identified as interval cancer in subsequent screening due to the poor sensitivity of screening for malignant calcifications (8). This is due to the low contrast and unclear boundaries on conventional images of breast mammograms (9). According to WHO, the survival probabilities of breast cancer patients may reach an astonishing number of 90% if the disease is identified and treated effectively in early stages.

Generally, in terms of detection, diagnosis, and treatment, many healthcare providers are faced with problems such as a lack of human resources and technological capabilities to deliver timely care to breast cancer patients (10). This problem worsens in developing and under-developed countries, where inexperienced radiologists are faced with a myriad number of mammogram images during screening. Therefore, the emergence of current computer-aided diagnosis (CAD) systems aids breast cancer diagnosis by allowing more comprehensive and objective analyses to be performed on many mammogram images. However, the CAD system is mostly based on hand-crafted features. The prognostic choice on the categorization of microcalcification clusters is mostly based on extracting useful handmade characteristics and then creating a highly discriminative classifier on top of them, which frequently yielded false results (11). Also, the installation of a sophisticated computer program in healthcare usually necessitates a multi-pronged strategy as it often involves political, economic, and social issues (12, 13).

The use of AI as an automated image classification tool has increased over the years as it allows automated disease diagnosis, characterization of histology, stage, or subtype, and patient classification based on therapeutic outcome or prognosis (14). Many types of diseases have incorporated the use of AI to form an automated prediction system. As such, the use of the Hippocampal Unified Multi-Atlas Network (HUMAN) algorithm to diagnose Alzheimer's disease (AD) (15). Current algorithms normally utilize transfer learning techniques or pre-trained CNNs to reduce the cost and time of training the network to allow automatic extraction of features at various levels of abstraction, features, and objects from raw images (16).

In the proposed work, we propose an end-to-end machine learning technique for automated breast cancer diagnosis using a pre-trained network to discriminate microcalcification, specifically a novel D-CNN architecture with adaptive transfer learning. In this study, curated Breast Imaging Subset of Digital Database for Screening Mammography (CIBS-DDSM) dataset from The Cancer Imaging Archive (TCIA) data portal which contains ROI images of digital mammography in grayscale will be utilized to facilitate training of the model. Our work utilizes CNN networks to automatically extract features of benign and malignant microcalcification instead of directing the machine to learn from locations identified *via* ^*^.csv files. A series of pre-processing algorithms are introduced to ensure the images were well prepared before beginning the process of feature extraction to enhance the accuracy of the model.

The primary contribution of the work involves; (i) proposing end-to-end machine learning architecture to diagnose breast cancer using microcalcifications' characteristics, (ii) performing pre-processing operations for the collected mammogram images before classification using deep learning algorithms, and (iii) proposing an adaptive transfer learning technique of CNN to build a breast cancer image classifier. The proposed work involves four state-of-the-art deep learning architectures such as ResNet38, ResNet50, VGG16, and AlexNet, and the performance of the models is compared to evaluate their performance.

### Related Works

The introduction of digital mammography images has made deep learning approaches for breast cancer diagnosis possible in recent years (17, 18). Significant research which involves the use of machine learning, specifically D-CNN-based supervised machine learning for microcalcification detection has been performed. CNNs are able to achieve higher detection accuracy as compared to CAD models by delivering quantitative analysis of suspicious lesions (19). Table 1 depicts the examples of studies that involve the classification of microcalcification of the breast into malignant and benign cases in recent years, including the model used and the accuracy achieved. Existing models of breast image classifiers for microcalcification detection are shown in Table 1. Based on Table 1, the highest accuracy for research breast image classifier involving VGG16 models is 94.3%, AlexNet is 88.6%, Resnet34 is 76.0%, and Resnet50 is 91.0%. Logic-based supervised learning such as Random Forest also managed to achieve an accuracy of 85.0% while Support Vector Machine (SVM) reached 95.8%.

**Table 1:** Models of breast image classifier for microcalcification detection.

**References**	**Base model**	**Type of image**	**Database**	**Accuracy**
Wang et al. (20)	Support vector machine (SVM)	Histopathology	Private	95.8%
Fadil et al. (21)	Random Forest	Mammography	DDSM	85.0%
Tsochatzidis et al. (22)	AlexNet	Mammography	CBIS-DDSM	75.3%
	VGG16			71.6%
	ResNet50 (training from scratch)			62.7%
	ResNet50 (pre-trained network)			74.9%
Xiao et al. (23)	2D ResNet34 with anisotropic 3D ResNet34	Digital breast Tomosynthesis (DBT)	Private DBT	76.0%
Li (24)	Modified VGG16	Mammography	Private, DDSM	90.0%
Khamparia et al. (25)	Hybrid ImageNet Modified VGG16	Mammography	DDSM	94.3%
	Modified VGG16			89.8%
	ResNet50			85.1%
	AlexNet			83.4%
Heenaye-Mamode Khan et al. (26)	ResNet50	Mammography	CBIS-DDSM, UPMC	88.0%
Cai et al. (27)	AlexNet	Mammography	Private	88.6%
Hekal et al. (28)	Modified AlexNet	Mammography	CBIS-DDSM	84.0%
	Modified ResNet50			91.0%

*Private = SunYat-sen University Cancer Center (Guangzhou, China) (SYUCC) and Nanhai Affiliated Hospital of Southern Medical University (NAHSMU) (Foshan, China)*.

As compared to learning algorithms such as SVM, CNN has gained its popularity due to higher accuracy and greater flexibility when it comes to tuning of hyperparameters. CNNs are feed-forward neural networks that are fully connected and are exceptionally good at lowering the number of parameters without sacrificing model quality. Since images have a high dimensionality as each pixel is considered a feature, it suits the capabilities of CNNs mentioned above.

As more models surfaced, accuracy has become one of the main aspects to compare the performance of models. Works of (22) highlighted that the accuracy for a pre-trained model is higher as compared to the scratch model. The accuracy for ResNet50 has achieved 62.7% for scratch model and 74.9% for pre-trained model respectively with the utilization of dataset from CBIS-DDSM. Ensemble modelling has also been observed in (24, 25, 27), where fusion or modification of existing models has been performed to produce a better model. For instance, the fusion of Modified VGG and ImageNet is observed in works of Khamparia et al. (25). This hybrid model enhances the performance of the model and achieved an astonishing accuracy of 94.3% in breast image classification (25). On the other hand, AlexNet based CNN model that is modified with multiple layer architecture and drop-out strategy together with the fusion of “off-the-shelf” model from ImageNet observed in (27) has demonstrated the ability to get robust and spatially invariant features, achieving an accuracy of 88.6% for morphologically filtered CNN feature.

Inspired by the promising results produced by the deep learning neural network, our research seeks to propose an end-to-end novel adaptive transfer learning convolutional neural network to discriminate microcalcifications of breast mammograms into benign or malignant cases. Most of the methods used were based on the Mammographic Image Analysis Society (MIAS) and InBreast dataset, which uses handcrafted features for machine learning. This research utilizes the CIBS-DDSM dataset obtained from TCIA data portal, which provides a higher resolution and number of images for machine learning to enhance the accuracy of diagnosis. Instead of training the model using a whole mammogram image, the model in this research is trained by using ROI images of calcifications, allowing the model to extract features from a focused area. The main goal of this research is to detect and categorize microcalcification as accurately as possible to aid radiologists to prepare the diagnosis report rapidly. The model is beneficial to be applied in a clinical setting.

## Materials and Methods

The proposed deep learning model is developed in Google Collab's platform with an OpenCV library of programming functions. Data acquisition is performed by downloading the breast mammography ROI images with microcalcification from the TCIA database. Micro-calcified images of the breast mammography were categorized into benign and malignant cases based on the information given in the ^*^.csv files from TCIA. Moving on, the downloaded images were pre-processed to remove artifacts and noises. Since the size of microcalcification is small and scattered in the mammogram, a conventional D-CNN model often failed to classify and often resulted in false positive or false-negative results. Therefore, we propose an end-to-end machine learning technique, which consists two stages of pre-processing technique, specifically implementation of artifacts removal to remove the existence of artifacts surfaced and filtering of images to lower the noise level of images prior to implementation of machine learning. The focus of work on enhancing quality of images were performed automatically upon identifying threshold value of breast region using Google Colab's platform. This step is crucially important to build and train a model with quality information of features extracted from the image itself.

**Figure 1:**
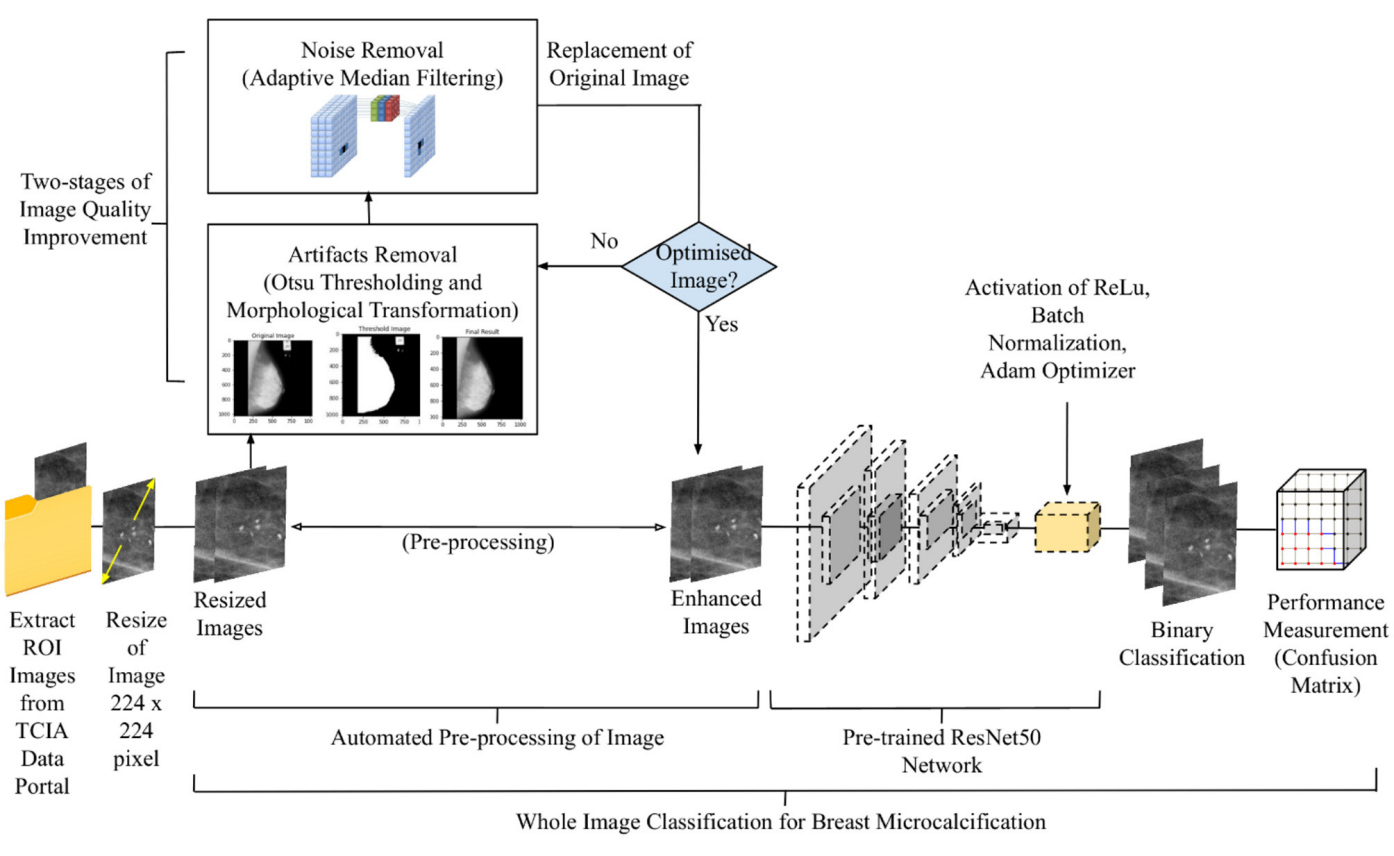
Workflow of the proposed design.

A D-CNN model is developed with finely tuned hyperparameters. To categorize the mammogram images into benign and malignant cases, a CNN model is utilized as a baseline. Transfer learning is used instead of training CNN from scratch. As such, different CNN models pre-trained with torch vision from the fastai library will be transferred to conduct the classification. To get the best possible result, hyperparameters such as the number of extra layers, learning rate, batch size, and epochs will be tuned. Finally, the confusion matrix will be utilized to assess the performance of the model to get the best possible accuracy. The overall algorithm for automated breast microcalcification classification is presented in Figure 1.

### Materials and Preparation of Dataset

The following are the materials needed for the work of this research:

1. Intel Core i7-4710 HQ, 3.5 GHz, 1 TB SSD, 4 GB RAM,
2. Google Colaboratory Platform (Python OpenCV language and fastai Library)
3. Breast Image dataset CIBS-DDSM from TCIA.

The CIBS-DDSM dataset of ROI microcalcification images for this research is obtainable from Cancer Imaging Archive (TCIA). The prepared dataset consists of 1,077 benign and 577 malignant ROI images in various sizes in DICOM format. Data Retriever software was installed to download radiological pictures from the TCIA Radiology Portal and was later fed into DICOM software to be saved in ^*^.jpeg format with a size of 224 × 224 to achieve uniformity in feature learning. The total number of images for benign and malignant as was multiplied by rotation at 90°, 180°, and 270°, resulting in 4,958 mammogram images for calcified benign ROI and 1,653 mammogram images for calcified malignant ROI. Table 2 shows the distribution of the dataset utilized in this study.

**Table 2:** Dataset distribution.

**Image**	**Calcified benign ROI**	**Calcified malignant ROI**
Original ROI image	1,077	577
Rotated at 90 degrees	1,077	577
Rotated at 180 degrees	1,077	577
Rotated at 270 degrees	1,077	577
Total number of images	4,958	1,653

### Pre-processing of Dataset

Before any pre-processing work was performed, the notebook on Google Collab was set to be under GPU Runtime to allow heavier computational work. Prior to training CNNs, the images will be pre-processed to remove the artifacts and improve the contrast by removing noise. *Otsu Segmentation Method* and *MorphologicalEx Method* presented by (29) were utilized to remove the artifacts that may be present at the image. *Otsu Segmentation Method* works on grayscale images and involves global thresholding or local thresholding to classify pixels values (30, 31). For instance, we denote mammogram image as function of *G*(*x, y*) and intensity value of *I* {*I* = 0, 1, 2, … *I*−1}. The variance of these two variables can be computed by using Equation (1).


(1) $$\begin{array}{l} \sigma_{m}^{2}=\theta_{1}^{\left(th\right)}\;\cdot \;\sigma_{1}^{2}{\left(th\right)}_{+\theta_{2}}\left(th\right)\;\cdot \;\sigma_{2}^{2}\left(th\right) \end{array}$$

whereby,


(2) $$\begin{array}{l} \theta_{1}\left(th\right)=\sum_{i=1}^{th}P\left(i\right) \end{array}$$

(3) $$\begin{array}{l} \theta_{2}\left(th\right)=\sum_{i=th+\;1}P\left(i\right) \end{array}$$

*P*(*i*) denotes the probability of gray-level *i* occurred, given as $P\;\left(i\right)=\frac{n_{i}}{n}$. In which, the number of pixels with a certain gray-level *I* is denoted by *i*. The image's total number of pixels is *n*. Threshold value *th*, which determines the class probability of pixels, is denoted as θ_1_ and θ_2_, and the mean of the class is calculated as *u*_1_and *u*_2_ as in Equations (4), (5) below. The threshold value that is predetermined earlier, *th*, which falls within the range of 0 < *th* < *I* will be utilized to divide the original mammogram image into two segments according to the intensity, which are [0, *th*] and [*th* + 1, *I*], where *I* is the maximum pixel value (255).


(4) $$\begin{array}{l} u_{1}\left(th\right)=\sum_{i=\;1}^{th}\frac{iP_{\left(i\right)}}{\theta_{1}\left(th\right)} \end{array}$$

(5) $$\begin{array}{l} u_{2}\left(th\right)=\sum_{i=th}\frac{iP_{\left(i\right)}}{\theta_{2}\left(th\right)}. \end{array}$$

The value of interclass variance and global mean-variance can then be computed by using Equations (6) and (7), respectively.


(6) $$\begin{array}{l} \sigma_{1}^{2}\left(th\right)=\sum_{i=1}^{I}{\left[1-u_{1}(th)\right]}^{2}\frac{P_{\left(i\right)}}{\theta_{1}\left(th\right)} \end{array}$$

(7) $$\begin{array}{l} \sigma_{2}^{2}\left(th\right)=\sum_{i=th+1}^{I}{\left[1-u_{2}(th)\right]}^{2}\frac{P_{\left(i\right)}}{\theta_{2}\left(th\right)}. \end{array}$$

The optimum threshold value is identified to achieve the best performance in distinguishing the target class from the background class, which is mostly utilized in mammography image binarization. Before executing the procedures for breast cancer detection segmentation and feature extraction, this thresholding approach is employed as a pre-processing technique (32, 33).

On the other hand, simple logical operations on local groupings of pixels, which is also defined as morphological operators are utilized in this research. Two of the main morphological operations used are dilation and erosion, which are shown in Equations (8) and (9), respectively (34). The binary image is denoted as *X* while the structuring element is denoted as *B*. The term *B*_*x*_ can be understood as translation of *B* by the vector *x*. Erosion reduces the size of an image by removing a layer of pixels from the inner and outer boundaries of regions. Dilation, on the other hand, has the reverse effect of erosion in that it adds a layer of pixels to both the inner and outer boundaries of regions. Many functions, such as opening and closing, are derived from these operators. When a picture is opened, it undergoes erosion and then dilation, and when it is closed, it undergoes dilation and then erosion (34).


(8) $$\begin{array}{l} X⊖B=\left\{x|B_{x}^{1}\subset X\;\right\} \end{array}$$

(9) $$\begin{array}{l} X⊕B=\left\{x|B_{x}^{2}\subset X^{c}\;\right\}. \end{array}$$

Adaptive median filter, mean filter and median filter were included in this research. The performance of filter was assessed according to Peak Signal to Noise Ratio (PSNR) and Mean Square Error (MSE). PSNR value is closely linked with MSE as it is computed based on MSE values, as in Equation (10).


(10) $$\begin{array}{l} PSNR=20{log}_{10}(\frac{{MAX}_{f}}{\sqrt{MSE}}) \end{array}$$

**Figure 2:**
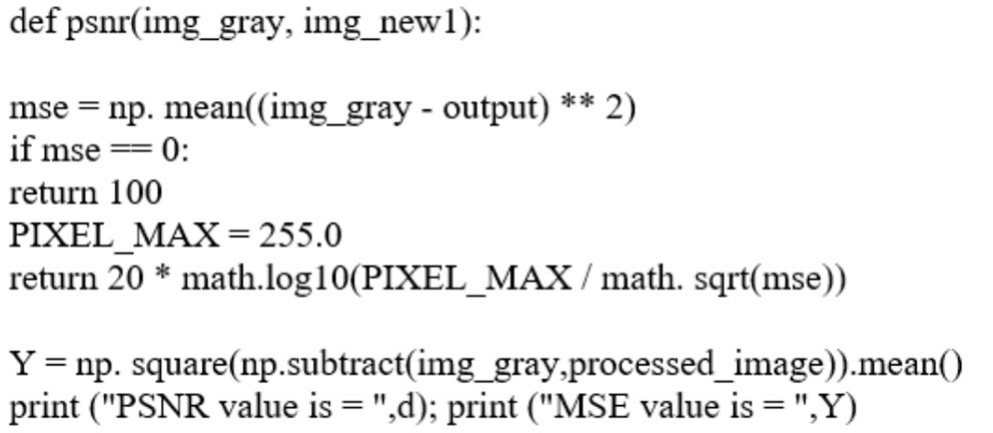
Code section for computing PSNR and MSE values based on filtered image and original image. “output” represents the finalized filtered image that will be used to compare with the original image, in this case is img_new1.

MAX_*f*_ is the maximum signal value that exists in the original image. Lower MSE indicates better filtration as MSE is the squared average of the “errors” between the actual image and the noisy image. The best filter will be selected based on the highest PSNR and lowest MSE value. The pseudocode of calculating MSE and PSNR value is shown in Figure 2.

Upon identifying the best performing filter, the filter will be applied to the images which has undergone artifacts removal process to further remove the noises of the image for clarity enhancement of the images, therefore completing the two-stages of optimization. The enhanced images will replace the original images to store the image in the same file location for machine learning. Before finalizing the two-stages of optimization process, the enhanced images will be inspected again to make sure the artifacts have been removed completely before proceeding to the next stage.

### Deep CNNs Architecture

Prior training, valid_pct () splits the dataset into training and testing sets at a particular ratio of 0.80 testing sets and 0.20 validation set. In total, there are 5,288 training images and 1,323 validation images. Data augmentation technique was implemented on the training set to avoid over-fitting by including get_transforms () function to increase the volume of the dataset by artificially producing new training data from the current data. Parameters of data augmentation is tabulated in Table 3.

**Table 3:** Parameters of data augmentation.

**Parameter**	**Function**	**Description**
Flipping	do_flip (), flip_vert ()	Flips the images at vertical and horizontal axis randomly
Zooming	max_zoom ()	Zooms the images at certain scale randomly
Rotating	max_rotate ()	Rotates the images at certain degree randomly
Lighting	max_lighting (), p_lighting()	Changes the contrast of image randomly controlled by max_lighting () with random probability ()

Hyperparameters were chosen manually in each set of tests to identify the best possible accuracy on binary classification. Hyperparameters that is tuned involves number of layers, learning rate, batch size as well as epoch. ADAM optimization algorithm was included to enhance the effectiveness of the model in to computing adaptive learning rate in complicated network architectures. In addition to that, ReLu is activated to prevent the computation required to run the neural network from growing exponentially. Batch Normalization is also activated to enable each layer of the network to conduct learning more independently by re-centering and re-scaling the layers' inputs to improve the speed and stability of the network.

**Figure 3:**
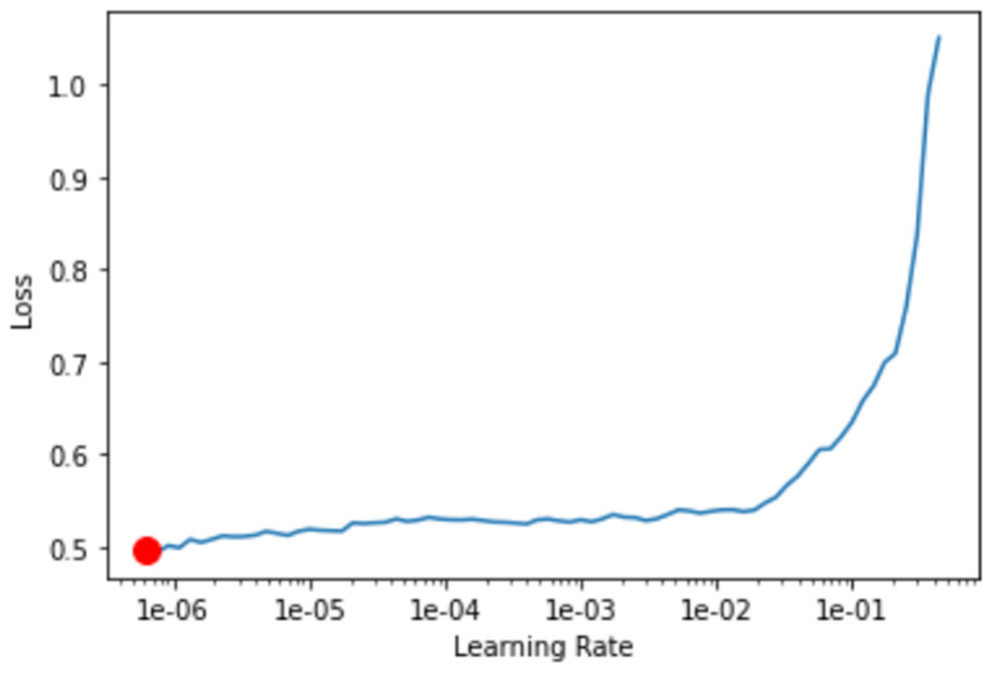
Learning curve plotted using learn.recorder.plot(). *Y*-axis depicts the learning loss while *X*-axis depicts the learning rate. Red dot shows the minimum gradient of the learning curve.

Pretrained network was downloaded from the fastai library using create_cnn (). The first layer of the model was trained by using learn.fit_one_cycle (). Later, the learning rate for the model was determined with the aid of learn.lr_find () and learn.recorder. plot (), which illustrates the learning curve of the model after training the first layer and suggests the lowest gradient of the learning curve. The example of learning curve plotted by using learn.recorder. plot () is shown in Figure 3.

Moving on, all layers of the model were unfreeze using learn.unfreeze () to allow more parameters to be trainable. The model undergoes training again with Cylindrical Learning Rate (CLR) using learn.fit_one_cycle (), but restrained on a cyclic learning rate using max_lr (). CLR enables the learning rate to fluctuate between appropriate minimum and maximum boundaries and is computationally cheap and eliminates the need to identify the ideal learning rate.

Upon running the number of epochs predetermined, the confusion matrix of the model on the validation set was plotted. The top losses of images during training were plotted with labels of “Prediction/Actual/Loss/Probability.” By the end of the training, the value for training loss, validation loss, error rate and accuracy were recorded.

### Performance Measurement

When it comes to evaluating the performance of the model, a confusion matrix is utilized. Four main parameters that are presented in a confusion matrix, which are: (i) True positive (TP) which shows the outcome of the model correctly predicts the benign cases, (ii) True negative (TN) which shows the outcome where the model correctly predicts the malignant cases, (iii) False positive (FP) which indicates the number of benign cases that are recognized as malignant cases by the model, and (iv) False negative (FN) which indicates the number of malignant case that are recognized as benign case by the model.

The values obtained from the confusion matrix will be further analyzed to compute additional parameters such as Recall, Precision, Specificity, Accuracy, F-1 score and Matthew Correlation Coefficient (MCC). MCC measures the performance of the parameters in the confusion matrix. The classifier produces a more accurate classifier if the MCC values trend more towards +1, and the reverse situation occurs if the MCC values trend more towards −1.


(11) $$\begin{array}{l} Recall=\frac{TP}{TP+FN} \end{array}$$

(12) $$\begin{array}{l} Precision=\frac{TP}{TP+FP} \end{array}$$

(13) $$\begin{array}{l} Specificity=\frac{TN}{TN+FP} \end{array}$$

(14) $$\begin{array}{l} Accuracy=\frac{TP+TN}{TP+TN+FP+FN} \end{array}$$

(15) $$\begin{array}{l} F1Score=\frac{2*Recall}{2*Recall+FP+FN} \end{array}$$

(16) $$\begin{array}{l} MCC=\frac{TP*TN\;-\;FP*FN}{\sqrt{(TP\;+\;FP)(TP\;+\;FN)(TN+FP)(TN\;+\;FN)}} \end{array}$$

## Results and Discussion

### Artifacts Removal

**Figure 4:**
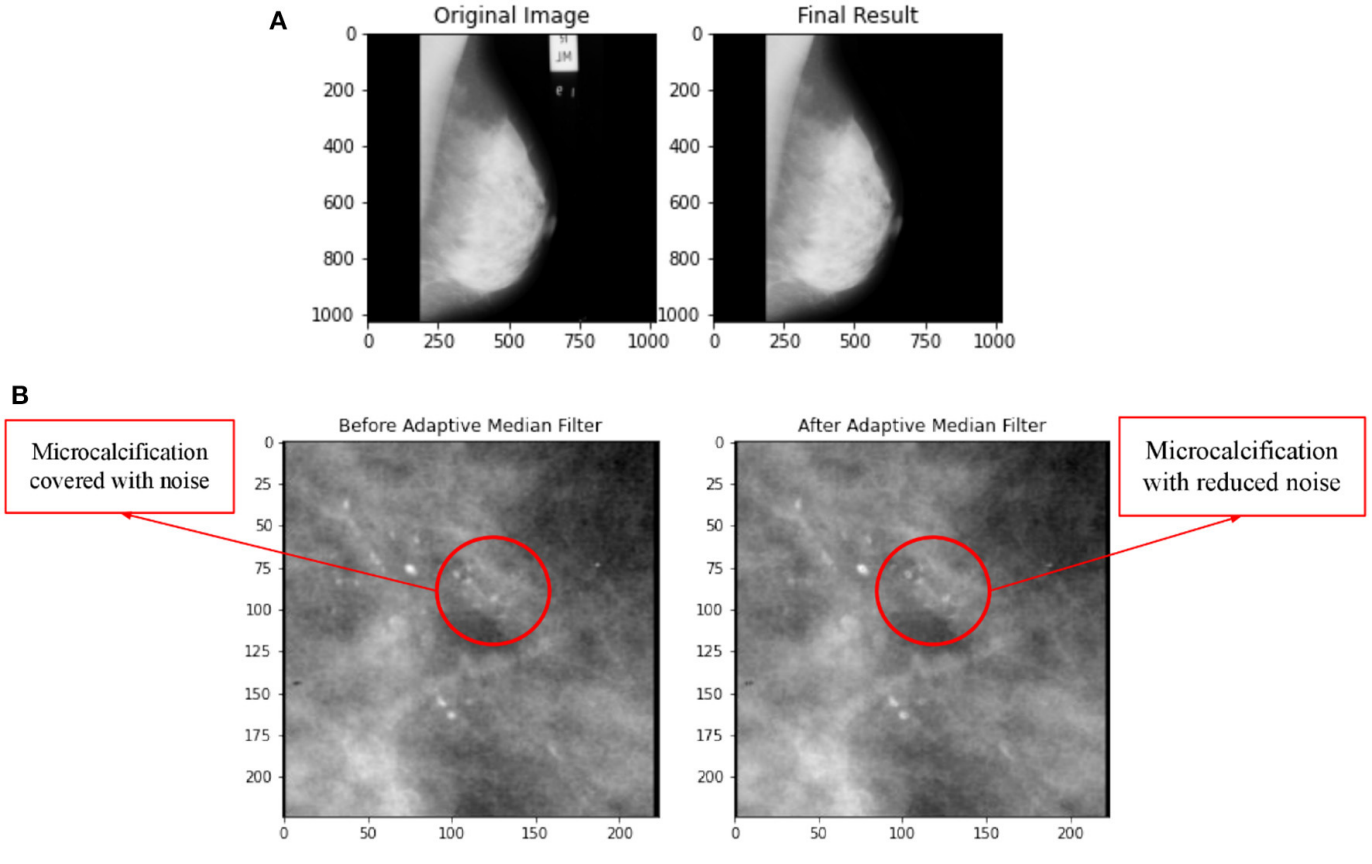
**(A)** Comparison on application of artifacts removal with implementation of *Otsu Segmentation Method* and *MorphologicalEx Method* for a sample of full breast mammogram image. **(B)** Comparison on before and after application of adaptive median filter.

Wedges and labels in the raw mammography picture may cause needless disruptions during the mass detection procedure (35). By manually looking at each ROI images of breast calcification downloaded from the TCIA database, the images were found to be free labelling artefacts. However, the algorithms for removal of artifacts were still conducted just in case there is hidden or unobvious artifact. In order to ensure that this section of coding works properly, a sample image of whole breast mammogram with obvious artifacts were imported and tested. The test result in Figure 4 shows successful removal of labelling artifacts with the whole breast mammogram image. Upon confirming the workability of the coding, the algorithm is then implemented to the ROI images in this study.

### Image Enhancement

In this research, three types of filters, namely adaptive median filter, mean filter, and median filter were applied on the same image and the MSE and PSNR value for each filter was computed to identify the best filter. Figure 4B shows a comparison of before and after application of adaptive median filter on breast mammogram image. The PSNR and MSE values for adaptive median filter, median filter and mean filter is tabulated in Table 4.

**Table 4:** PSNR and MSE values for adaptive median filter, median filter and mean filter.

**Parameter**	**Adaptive median filter**	**Median filter**	**Mean filter**
PSNR	42.3863	37.5911	36.9511
MSE	3.7536	11.3233	13.1213

By referring to Table 4, value for MSE is lowest for adaptive median filter, indicating that the error difference between the original image's values and the degraded image's values for adaptive median filter is the least among all three types of filters. Similar to (36, 37), comparison for adaptive median, mean and median filter for breast mammogram images were reported and the authors had concluded that adaptive median filter is the best filter for noise reduction since the quality of the image produced is much superior. Hence, this research utilizes adaptive median filter for image enhancement of breast microcalcification images.

### CNN Model Architecture

Tables 5–8 show the output of VGG16, ResNet34, AlexNet and Resnet50 models respectively. Identifying ideal batch size for CNNs is important as it helps the network to reach maximum accuracy in the quickest possible time, particularly for complicated datasets, such as a medical picture dataset (38). Results obtained from this study demonstrates that with learning rate and epochs remains, the accuracy of the model increases when the number of batch sizes increases from 32 to 64. In Table 7, the increase in batch size from 32 to 64 in Test 10 and Test 11 has resulted in increase in accuracy with an additional value of 4.67%. Findings from this research also implies that the larger the batch size, the greater the network accuracy, implying that batch size has a significant influence on CNN performance.

**Table 5:** Output of VGG16 model.

**Test**	**Batch size**	**Learning rate**	**Epoch**	**Training loss**	**Validation loss**	**Error rate**	**Accuracy**
1	32	8e-6,1e-4	15	42.7083	43.9302	23.4493	76.5507
2	64	8e-6,1e-4	15	76.4934	50.4612	22.4917	77.5083
3	64	8e-6,1e-4	30	26.2982	45.7910	18.0787	81.9213
4	32	2e-6,1e-3	15	30.7861	32.0147	16.4522	83.5478
5	64	2e-6,1e-3	15	25.4205	25.4679	10.9682	89.0318
6	64	2e-6,1e-3	30	7.5000	8.4696	3.0257	96.9743

**Table 6:** Output of ResNet34 model.

**Test**	**Batch size**	**Learning rate**	**Epoch**	**Training loss**	**Validation loss**	**Error rate**	**Accuracy**
7	32	8e-6,1e-4	15	42.2252	43.1934	21.4070	78.5930
8	64	8e-6,1e-4	15	41.1351	42.8464	21.5582	78.4418
9	64	8e-6,1e-4	30	12.6166	36.0723	16.3888	83.6112
10	32	2e-6,1e-3	15	35.0093	30.7748	14.2965	85.7035
11	64	2e-6,1e-3	15	26.2728	26.9305	10.8926	89.1074
12	64	2e-6,1e-3	30	7.6075	9.5925	2.6475	97.3525

**Table 7:** Output of AlexNet model.

**Test**	**Batch size**	**Learning rate**	**Epoch**	**Training loss**	**validation loss**	**Error rate**	**Accuracy**
13	32	8e-6,1e-4	15	52.147	48.5449	26.0968	73.9032
14	64	8e-6,1e-4	15	49.9790	46.5579	25.416	74.5840
15	64	8e-6,1e-4	30	42.8953	44.6564	24.2814	75.7186
16	32	2e-6,1e-3	15	46.3035	42.8736	22.1044	77.8956
17	64	2e-6,1e-3	15	44.2203	42.6651	22.0121	77.9879
18	64	2e-6,1e-3	30	39.0666	35.3782	16.9440	83.0560

**Table 8:** Output of ResNet50 model.

**Test**	**Batch size**	**Learning rate**	**Epoch**	**training loss**	**Validation loss**	**Error rate**	**Accuracy**
19	32	8e-6,1e-4	15	39.0362	41.5517	20.5749	79.4251
20	64	8e-6,1e-4	15	35.1833	40.6826	19.5159	80.4841
21	64	8e-6,1e-4	30	21.1929	36.9642	14.2965	85.7035
22	32	2e-6,1e-3	15	20.5363	37.8652	15.5068	84.4932
23	64	2e-6,1e-3	15	29.6796	24.4782	10.6657	89.3343
24	64	2e-6,1e-3	30	10.8362	5.8117	2.4206	97.5794

**Figure 5:**
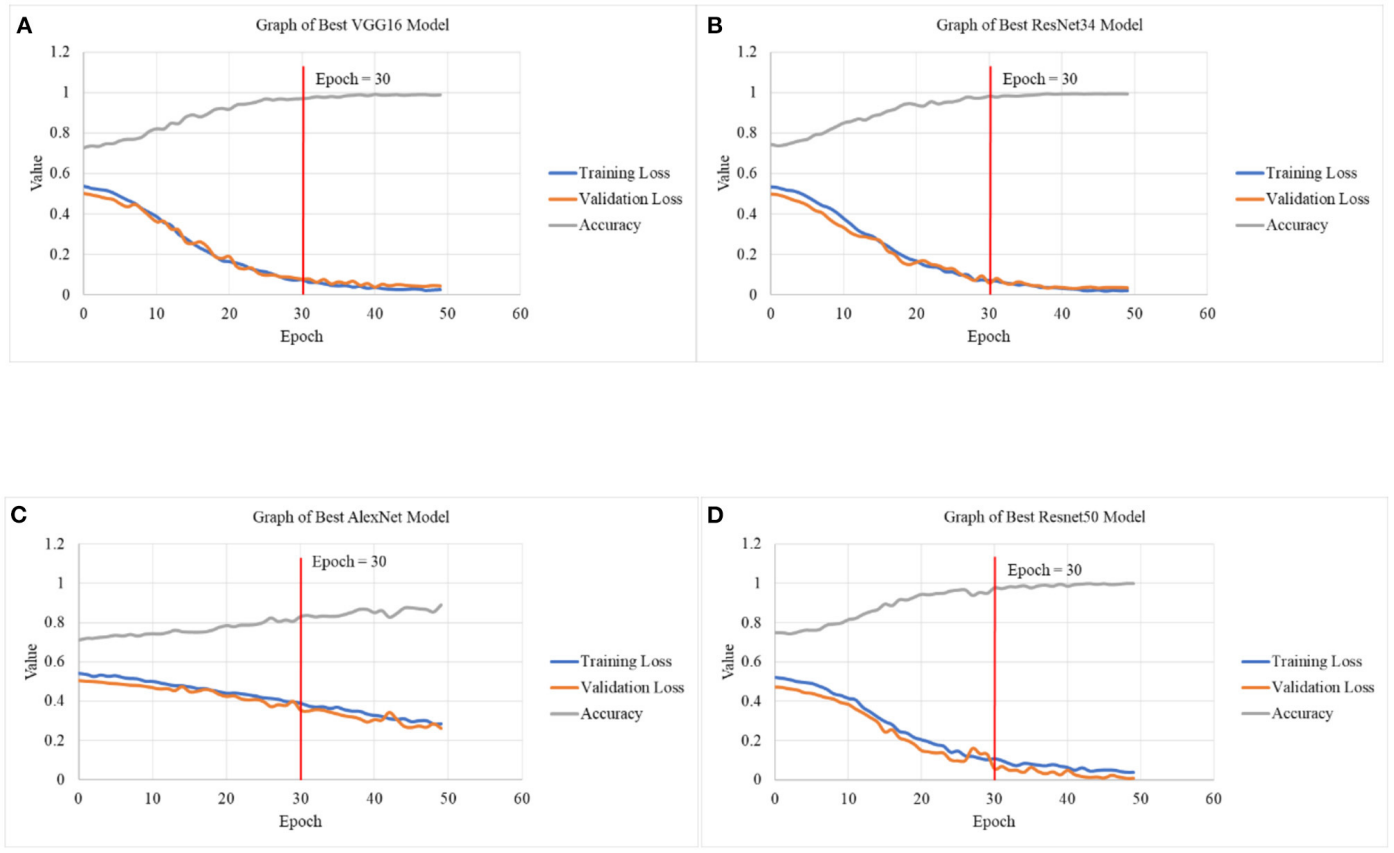
Graphical illustration of CNN models in terms of Training Loss, Validation Loss and Accuracy in **(A)** graph of best VGG16 model, **(B)** graph of best ResNet34 model, **(C)** graph of best AlexNet model, and **(D)** graph of best ResNet50 model.

Figure 5 depicts the graphical illustration of CNN models in terms of Training Loss, Validation Loss and Accuracy for different models. Graphs obtained from this study suggests better accuracy was achieved with smaller learning rates of 2e-6,1e-3 as compared to 8e-6,1e-4. With number of epochs increases, the accuracy tends to increase as well. In Table 5 Test 5 and 6, with learning rate of 2e-6,1e-3, the accuracy of VGG16 has managed to reach 96.9743% for 30 epochs as compared to 89.0318% for 15 epochs. Test 17 and 18 also demonstrates the same characteristic with an increase of accuracy from 77.99 to 83.06%, about a 6.50% difference with increase of 15 to 30 epochs.

By referring to Figure 5, upon reaching 30 epochs, the losses and accuracy starts to flatten out, suggesting overfitting. Overfitting occurs when the network begins to overfit the data and the error on the validation set will soon begin to rise on a regular basis. This is where training should be terminated (39, 40). Therefore, the number of epochs for all the models is fixed at 30. In addition to that, the training and validation loss at 30 epochs is not increasing nor achieving linearity before minimal loss is achieved, suggesting that the result is not overfitting.

### Comparison of Models With Existing Work

As deep learning becomes more popular, more researchers created new architectures with deeper CNN in radiomics of mammographic imaging to improve breast cancer diagnosis (41). VGG net requires much more parameters to thoroughly evaluate its performance. In (30, 31), the use of VGG16 was modified to classify microcalcification images into benign or malignant cases from the DDSM database and obtained accuracy of 94.3 and 87.0%, respectively. Study of (33) utilized AlexNet and managed to achieve an accuracy of 79.1% upon utilizing 10-fold cross validation technique with 300 epochs and learning rate of 0.01 based on 900 images from SYUCC and NAHSMU database. In this research, the technique of cross validation was not performed, but the accuracy achieved in AlexNet is much higher, reaching 83.1% with just 30 epochs. The difference in the result might be due to the different database of images that was used. For instance, this research utilizes ROI calcification images of CIBS-DDSM database which provides higher resolution. Also, the learning rate that was used in this study is much smaller. Study of (42) highlights that smaller learning rate can frequently increase generalization of accuracy substantially. A slower learning rate may allow the model to learn a set of weights that is more optimum or even globally optimal. This might explain why smaller learning rates may also be able to produce models with higher accuracy.

Study of (34) classified 1,852 calcification images of CIDB-DDSM database into CNN pretrained models of modified AlexNet and ResNet50, of which the FC8 layer in AlexNet or FC1000 layer in ResNet50 is replaced with a shallow classifier (SVM). With 20 epochs, the accuracy for breast microcalcification for Resnet50 has managed to reach 91% while AlexNet has reached 90%. Although the accuracy for the AlexNet model in this study was lower (83.1%), the accuracy for Resnet50 managed surpassed with a value of 97.6%. Modified ResNet50 was also observed in (26, 32, 43), with (43) achieving the highest accuracy of 90.3% upon utilizing 354 images from Inbreast dataset. The Resnet50 model in this study is able to surpass existing work with accuracy value of 97.6%. The main difference between the models is the image that is fed to the machine for training. For instance, this research directly utilizes ROI calcification images of CIBS-DDSM database, which enables the machine to learn the features of malignant and benign calcified cases accurately.

The use of Resnet34 in breast microcalcification can be observed in the study of (23), where the authors utilized 2D Resnet34 together with anisotropic 3D Resnet to classify 495 Digital Breast Tomosynthesis (DBT) microcalcification images and reached an accuracy value of 76.0%. The model of Resnet34 in this study is able to provide a significantly higher accuracy value, which is 97.4%, probably due to the large number of images (6,611 images) utilized for machine learning, of which is 13 times larger than the study of (23).

**Figure 6:**
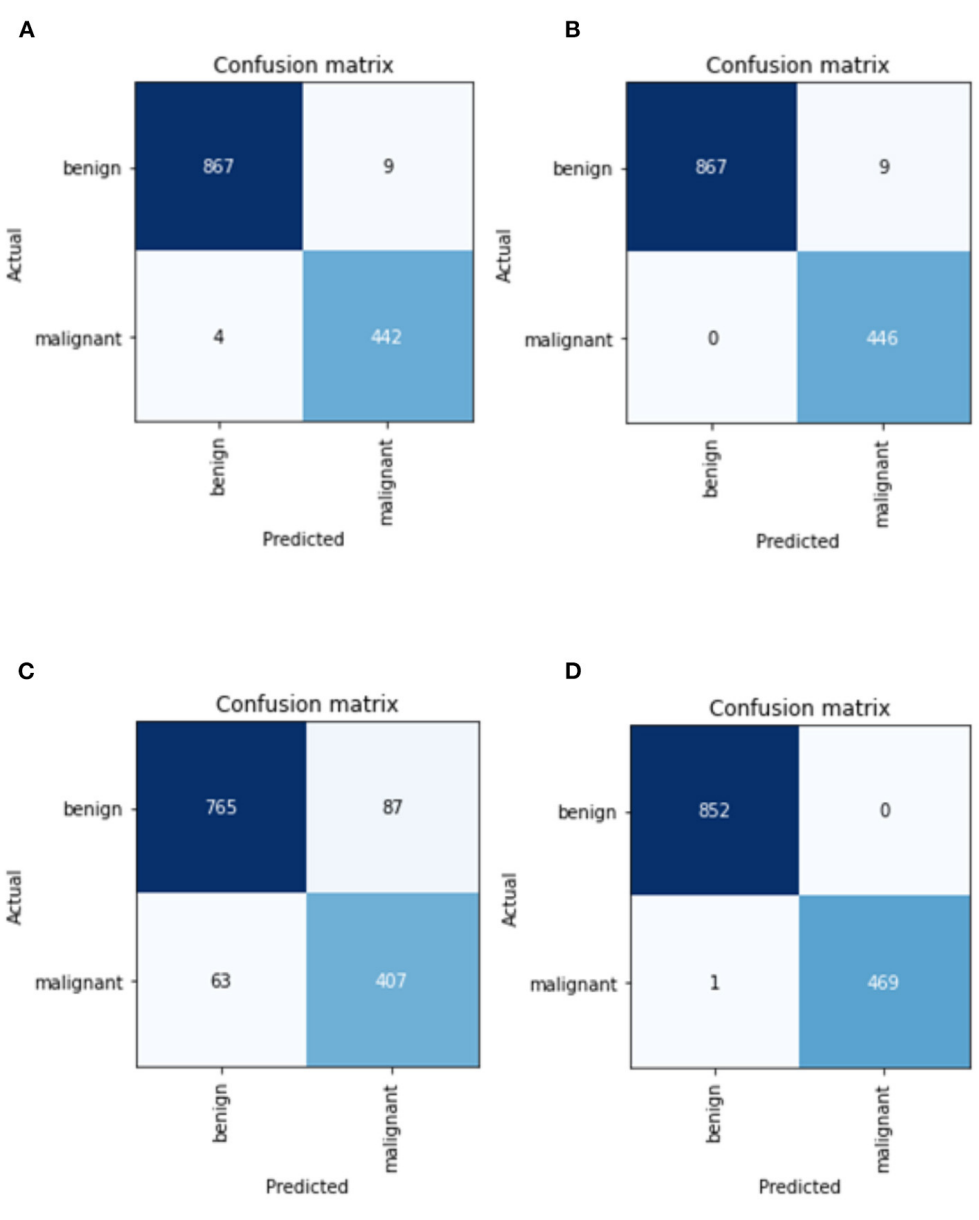
Confusion matrix of CNN models in **(A)** best VGG16 model, **(B)** best ResNet34 model, **(C)** best AlexNet model, and **(D)** best ResNet50 model.

Figure 6 depicts the confusion matrix of CNN models. Overall, the AlexNet model has the highest percentage of both falsely classified benign and falsely classified malignant cases, which is 11.37% and 15.48%, respectively. The performance of the Resnet50 is considered as the best because it only has 1 misclassified image over 1,322 images, while Resnet34 has a total of nine misclassified images. For the case of VGG, it has a total of 13 misclassified images. Based on the values obtained in the confusion matrix, calculation for additional performance measurement was performed and tabulated in Table 9.

**Table 9:** Additional performance measurement for best Resnet34, Resnet50, VGG16 and AlexNet model.

**Architecture**	**Recall**	**Precision**	**Specificity**	**Accuracy**	**F-1 Score**	**MCC**
Resnet34	1.0000	0.9897	0.9802	0.9932	0.1818	0.8950
**Resnet50**	**0.9988**	**1.0000**	**1.0000**	**0.9992**	**0.6664**	**0.9983**
VGG16	0.9954	0.9897	0.9800	0.9902	0.1328	0.9781
AlexNet	0.9239	0.8979	0.8239	0.8865	0.0122	0.7558

*Bold values indicates the model with the best performance*.

Based on Table 9, Resnet50 has the highest precision, specificity, and accuracy, while ResNet 34 model has the highest Recall, which is also referred to as True Positive Rate or Sensitivity. Result from this study suggests that the performance by ResNet model outperforms VGG and AlexNet models. ResNet50 also has the highest F1-score (0.6664), which indicates how accurate a model is on a given dataset. MCC, can be considered as the most credible statistical metric since it is only high if all four confusion matrix categories are correctly predicted. From this study, Resnet50 is able to achieve the highest score of MCC with a value of 0.9983.

In a summary, an automated microcalcification detection in mammography for early breast cancer diagnosis using deep learning techniques has been successfully developed. Collected greyscale mammogram images had undergone pre-processing operations which includes conversion of images from DICOM to ^*^.jpeg format, resizing to 224 × 224 pixels, removal of artifacts, and image enhancement by application of adaptive median filter. Transfer learning technique for CNN architectures was employed and result shows that ResNet50 achieves the highest accuracy with a value of 97.58%, followed by ResNet34 of 97.35%, VGG16 of 96.97% and finally AlexNet of 83.06%. The main limitation with current work is the possibility of the machine to remember the repeated patterning of the dataset for classification into benign or malignant cases *via* implementation of data augmentation. Resizing of ROI images might also result in data compression and loss of useful features or information of the image.

## Conclusions

Our proposed work has built an end-to-end novel adaptive transfer learning convolutional neural network that has shown ability to discriminate microcalcifications of breast mammograms into benign or malignant cases. ROI breast images were acquired from CIBS-DDSM database to obtain a higher resolution image of breast mammogram. The selection of quality datasets, abundancy of images for training, as well as tuning of hyperparameters are all important in improving the accuracy of the models. We have also shown a quantitative analysis on the effectiveness of three filters, namely adaptive median, median and mean filter in noise removal of breast microcalcification mammogram images by calculating the MSE and PSNR value. As compared to traditional method of feature extraction which uses coordinates to identify the location of microcalcification, we have successfully automize the model to identify the characterization of benign and malignant microcalcification patterns. All CNN models that were trained shows powerful ability to discriminate benign and malignant microcalcification, with ResNet50 achieving the highest accuracy of 97.58%.

Breast cancer is a significant threat to women or men all over the world and improving the existing state of breast cancer detection systems is definitely a critical challenge. Findings from this study will be able narrow the gap of findings for CNNs models which were mostly tailored for binary classifier that focuses solely on breast microcalcification classification by providing a comparative comparison beginning from datasets that is utilized, pre-processing algorithms that are included, up to the algorithms utilized during machine learning. In addition, this study will also be able to aid research in developing a competent binary classification model by providing a comprehensive approach to the recent results on different CNN models in breast microcalcification detection. In future, different sources of breast images could be incorporated, such as 3D mammogram images, in order to identify and compare the effectiveness of the model in classifying different sources of microcalcification images. K-fold cross validation could also be incorporated in the algorithm to combine metrics of prediction fitness to get a more accurate estimate of model prediction performance.

## Data Availability Statement

Publicly available datasets were analyzed in this study. This data can be found at: https://www.cancerimagingarchive.net.

## Author Contributions

YL and KH designed and developed the algorithm as well as major contributors to the article writing. KL, NM, and MA performed the comparison analysis and checked all the synthesized data. All authors approved the final version to be submitted for publication.

## Funding

The project was funded by University Malaya Research Grant Faculty Programme (RF010-2018A).

## Conflict of Interest

The authors declare that the research was conducted in the absence of any commercial or financial relationships that could be construed as a potential conflict of interest.

## Publisher's Note

All claims expressed in this article are solely those of the authors and do not necessarily represent those of their affiliated organizations, or those of the publisher, the editors and the reviewers. Any product that may be evaluated in this article, or claim that may be made by its manufacturer, is not guaranteed or endorsed by the publisher.
